# Two new records of the genus *Trioxys* (Hymenoptera, Braconidae, Aphidiinae) parasitic on bamboo aphids from South Korea

**DOI:** 10.3897/BDJ.12.e118599

**Published:** 2024-03-14

**Authors:** Sangjin Kim, Juhyeong Sohn, Hyojoong Kim

**Affiliations:** 1 Kunsan National University, Gunsan, Republic of Korea Kunsan National University Gunsan Republic of Korea

**Keywords:** DNA barcoding, natural enemy, parasitoid wasps, systematics, taxonomy

## Abstract

**Background:**

The genus *Trioxys* Haliday, 1833 consists of more than 80 species worldwide with three species being recorded in South Korea. In this study, we report the first observation of the two additional species, *T.liui* Chou & Chou, 1993 from *Takecallisarundinariae* (Essig, 1917) on *Phyllostachysbambusoides* Siebold & Zucc., 1843 and *T.remaudierei* Starý & Rakhshani, 2017 from *T.taiwana* (Takahashi, 1926) on *Sasaborealis* (Hack.) Makino & Shibata, 1901.

**New information:**

*Trioxysliui and T.remaudierei* are described and reported with phototographs of the diagnostic morphological characters and the mitochondrial *cytochrome c oxidase subunit I* (*COI*) data (barcode region) and Bayesian tree of the phylogenetic analysis amongst the closely-related taxa are provided.

## Introduction

The genus *Trioxys* Haliday, 1833 (Hymenoptera, Braconidae, Aphidiinae) consists of more than 80 known species around the world (Rakhshani et al. 2012, Yu et al. 2016, Rakhshani et al. 2017, Čkrkić et al. 2021, Davidian 2021, Petrović et al. 2021), with three species being recorded in South Korea (National Institute of Biological Resources 2022). As a key character for classification to the two morphologically similar genera, *Trioxys* Haliday, 1833 and *Binodoxys* Mackauer, 1960, a pair of accessory prongs are evident on the abdominal sternite. The former has only a spiracular (primary) tubercle, while the latter has not only a spiracular tubercle, but also has a secondary tubercle (Mackauer 1961, Starý 1981).

The plant subfamily Bambusoideae Bambusoideae Luerss., (1893) (Cyperales, Poaceae), commonly known as Bamboo, consists of 120 genera with more than 1600 species in the world (Soreng et al. 2017) and five genera with 13 species being recorded in South Korea (National Institute of Biological Resources 2022). Bamboo, an evergreen perennial flowering plant, is represented by three main species in Korea: *Phyllostachysbambusoides* Siebold & Zucc., 1843, P.nigravar.henonis (Mitford) Stapf ex Rendle, 1904 and *Pseudosasajaponica* (Siebold & Zucc. ex Steud.) Makino ex Nakai, 1920, these being the most widely distributed in the region (Kelchner 2013, Zhao et al. 2019).

The genus *Takecallis* Mastumura, 1917 (Hemiptera, Aphididae, Calaphidinae) consists of eight valid species in the world (Remaudière and Remaudière 1997, Lee and Lee 2018, Wieczorek and Sawka-Gądek 2023) with five species recorded in South Korea (National Institute of Biological Resources 2022). *Takecallis* species was regarded as a pest in various bamboos, *Arundinaria* spp., *Bambusa* spp., *Dendrocalamus* spp., *Phyllostachys* spp., *Pleioblastus* spp., *Pseudosasa* spp., *Sasa* spp. and *Yushania* sp. (Quednau 2003, Qiao and Zhang 2004). In this study, we provide the dignostic characters for two species, *Trioxysliui* and *T.remaudierei*, from the *Takecallis* species in bamboo spp. and analyse their phylogenetic amongst closely-related congeneric species, using the *COI* barcode region.

## Materials and methods

### Field and Taxonomic works

Samples were collected by searching for *Takecallis* mummies (*T.taiwana* (Takahashi, 1926) and *T.arundinariae* (Essig, 1917)) on various bamboo species (*Phyllostachysbambusoides* and *Sasaborealis*). Leaves containing mummified aphids were then collected and placed in a clean insect breeding dish (SPL Life Sciences, Korea). To ensure a sufficient number of samples, these dishes were kept in the laboratory at room temperature. The emergence of parasitoid wasps was monitored daily and they were collected using an insect aspirator. Subsequently, the collected wasps were preserved in 80% ethyl alcohol at -19℃.

Morphological identification of *Trioxys* species was referred from Chou and Chou (1993), Davidian (2007), Yu et al. (2016), Das and Chakrabarti (2017), Rakhshani et al. (2017), Rakhshani et al. (2020) and that of *Takecallis* species from Lee and Lee (2018) and Wieczorek and Sawka-Gądek (2023). Terminology of morphological characters for the Aphidiinae follows Wharton et al. (1997) (for the venation of wing) and Takada (1968). We first performed morphological sorting of similar phenotypes and labelling of this sample using a stereomicroscope (OLYMPUS SZX16, Leica M205C), after which DNA extraction was performed.

After conducting both morphological and molecular identification, measurements of unrecorded species were carried out. A LEICA DMC2900 digital camera and a LEICA M205 C microscope (Leica Geosystems AG) were utilised for photography and characterisation. Multiple pictures were taken at various heights using multifocusing technology. LAS V4.11 (Leica Geosystems AG) and HeliconFocus 7 (Helicon Soft) software were used for the stacking process. After the stacking procedure, illustrations were generated using Adobe Photoshop CS6. LAS V.4.11 (Leica Geosystems AG) was utilised to determine the shape of the specimens (Berkovitch et al. 2009).

### Molecular analysis

Total genomic DNA extraction was performed using a LaboPass Tissue Kit (COSMOgenetech, Korea) following the manufacturer’s protocol. To preserve a morphologically complete specimen, the DNA extraction method was slightly modified from the “freezing method” used by Yaakop et al. (2013). In the original protocol, the sample was incubated for 30 minutes at 56°C with 200 μl of TL buffer + 20 μl of proteinase K. In the slightly modified DNA extraction methods, a 2 hour incubation period at the same temperature was used. Genomic DNA was extracted individually from each sample.

The target site for molecular identification was the front partial region of mitochondrial *COI*, a 658-bp fragment, amplified using primers, LCO1490 (forward) 5’-GGTCAACAAATCATAAAGATATTGG-3’ and HCO2198 (reverse) 5'-TAAACTTCAGGGTGACCAAAAAATCA-3’ (Folmer et al. 1994), with AccuPower PCR PreMix (Bioneer Corp., Daejeon, Korea). Polymerase chain reaction (PCR) amplification was conducted with 20 ml of a reaction mixture consisting of 3 ml of DNA extract, 2 ml of primer and 15 ml of ddH_2_O. It was carried out as follows: denaturation for 5 min at 95℃; 4 cycles of 20 s at 95℃, 30 s at 55℃ (decreasing incrementally by 2℃ per cycle) and 40 s at 72℃, 31 cycles of 20 s at 95℃, 30 s at 48℃ and 40 s at 72℃; and final extension at 72℃ for 5 min. PCR products were visualised by electrophoresis on agar gel and, if a band existed, we commissioned Macrogen (Daejeon, Korea) for purification and sequencing analysis.

Using MEGA version 7.0 (Kumar et al. 2016), sequences were aligned by ClustalW default settings and their frame-shifts checked to avoid pseudogenes. Alignments were translated to amino acids using MEGA version 7.0. We calculated sequence divergences using the ‘*p*-distance’ model commonly with 1,000 bootstrapping replications and complete deletion in data gaps.

A phylogenetic tree was constructed with the Bayesian method using BEAST2 (Bouckaert et al. 2014). To produce dated phylogenies, we used an optimised relaxed clock model (Drummond et al. 2006) in BEAUti and other options were set at default. MCMC analysis was performed and checked using Tracer, DensiTree. After that, we constructed the consensus tree using TreeAnnotator with posterior probability limit 1.0 setting.

Molecular identification was based on Ratnasingham and Herbert (2007). The front partial region 658 bp of the *COI* fragment was sequenced from *T.liui* and *T.remaudierei* and deposited in GenBank. Altogether, 14 sequences of six species, containing the outgroup, were retrieved from GenBank and BOLD (http://www.boldsystems.org) and were used to compare them with *T.liui* and *T.remaudierei* (Table 1).

## Taxon treatments

### Trioxys liui

Chou & Chou, 1993

E24207D1-7A28-5DD0-A101-6E2156743BD0

PP373116

PP373117

*Trioxysliui* Chou & Chou, 1993 - Chou and Chou (1993): 375-378.
*Trioxys* sp. Starý and Schlinger (1967), 1967: 127.
*Trioxysbambusa* Liu, 1975 - Liu (1975): 69 (nomen nudum).

#### Materials

- **Type status:** Other material. **Occurrence:** individualCount: 6; sex: female; lifeStage: adult; occurrenceStatus: present; preparations: in 80% ethanol and dry-specimen; occurrenceID: 1A829D92-1D9D-5A03-BB52-E23958988DE4; **Taxon:** scientificName: Trioxysliui Chou & Chou, 1993; kingdom: Animalia; phylum: Arthropoda; class: Insecta; order: Hymenoptera; family: Braconidae; genus: Trioxys; specificEpithet: liui; taxonRank: species; scientificNameAuthorship: Chou & Chou, 1993; **Location:** higherGeography: East Asia; country: South Korea; countryCode: KR; stateProvince: Jeollabuk-do; municipality: Gunsan-si; locality: 290-2, Singwan-dong; **Identification:** identifiedBy: Sangjin Kim, JuHyeong Sohn, Hyojoong Kim; **Event:** eventDate: 08-11-2022; year: 2022; month: 11; day: 8; **Record Level:** institutionCode: KSNU; basisOfRecord: preserved specimen

#### Description

**Figure 1.**
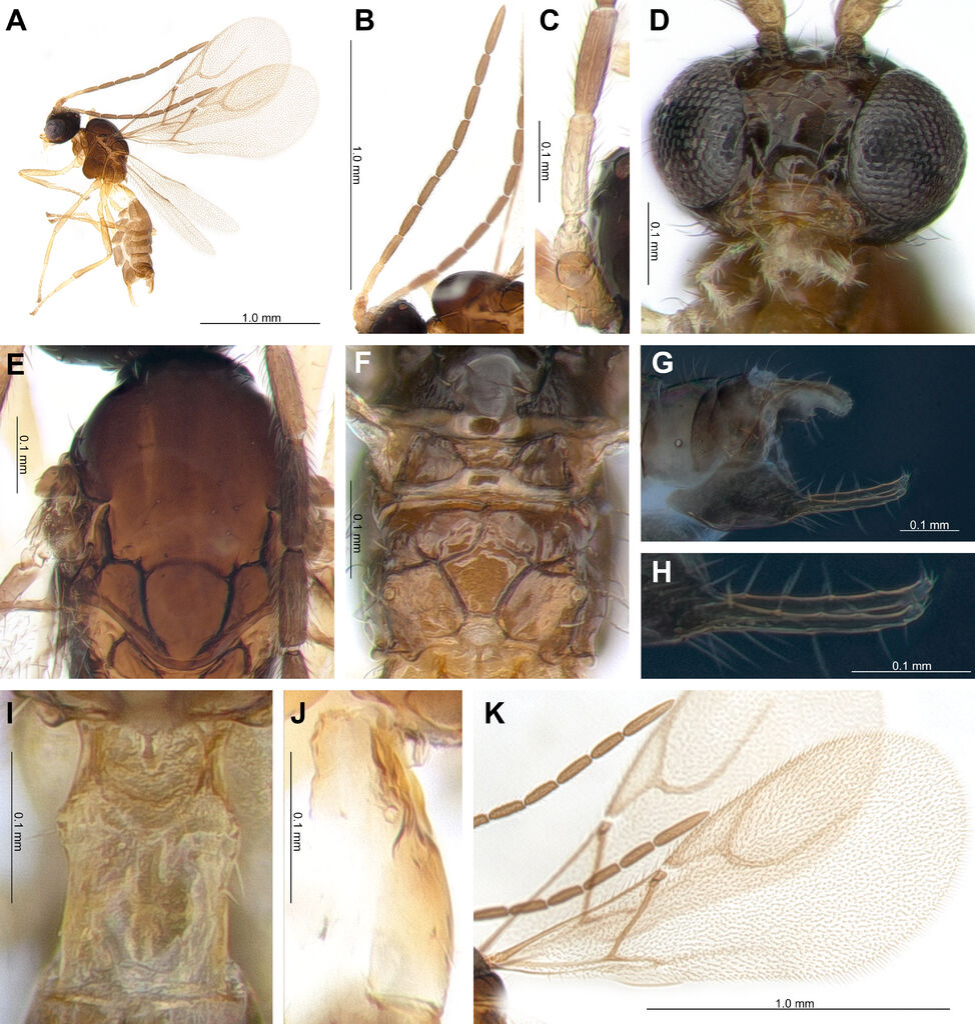
*Trioxysliui*: **A** Habitus; **B** Antenna; **C** F1 and F2; **D** Head; **E** Mesoscutum; **F** Propodeum; **G** Ovipositor; **H** Prong; **I** Dorsal view of petiole; **J** Lateral view of petiole; **K** Forewing.

**Female.** Length of body about 1.99 mm (Figure Fig. 1A). Length of forewing 1.67 mm (Fig. 1K).

**Head.** Eyes oval, sparsely setose. Tentorial index 0.20 (Fig. 1D). Clypeus oval with 6 setae. Malar space 0.09 times as long as longitudinal eye diameter. Antenna 11-segmented (Fig. 1B). F1 equal with F2 (Fig. 1C). F1 and F2 4.1 and 3.4 times as long as their width at the middle, respectively. F1 and F2 are with two and three longitudinal placodes, respectively. Maxillary palp with four palpomeres, labial palp with two palpomeres. Ratio of eye to temple in dorsal view 0.7. Face width/height ratio 1.3 (Fig. 1D).

**Mesosoma.** Mesoscutum with notaulices on anterior part, dorsal surface smooth, scarcely setose (Fig. 1E). Head width/mesoscutum width ratio 1.4. Propodeum areolated, areola length/width ratio subequal (1.01×) (Fig. 1F). Pterostigma 3.6 times as long as width. Ratio of pterostigma length to R1 vein (= metacarpus) length 1.4. r and RS veins extended (Fig. 1D).

**Figure 2.**
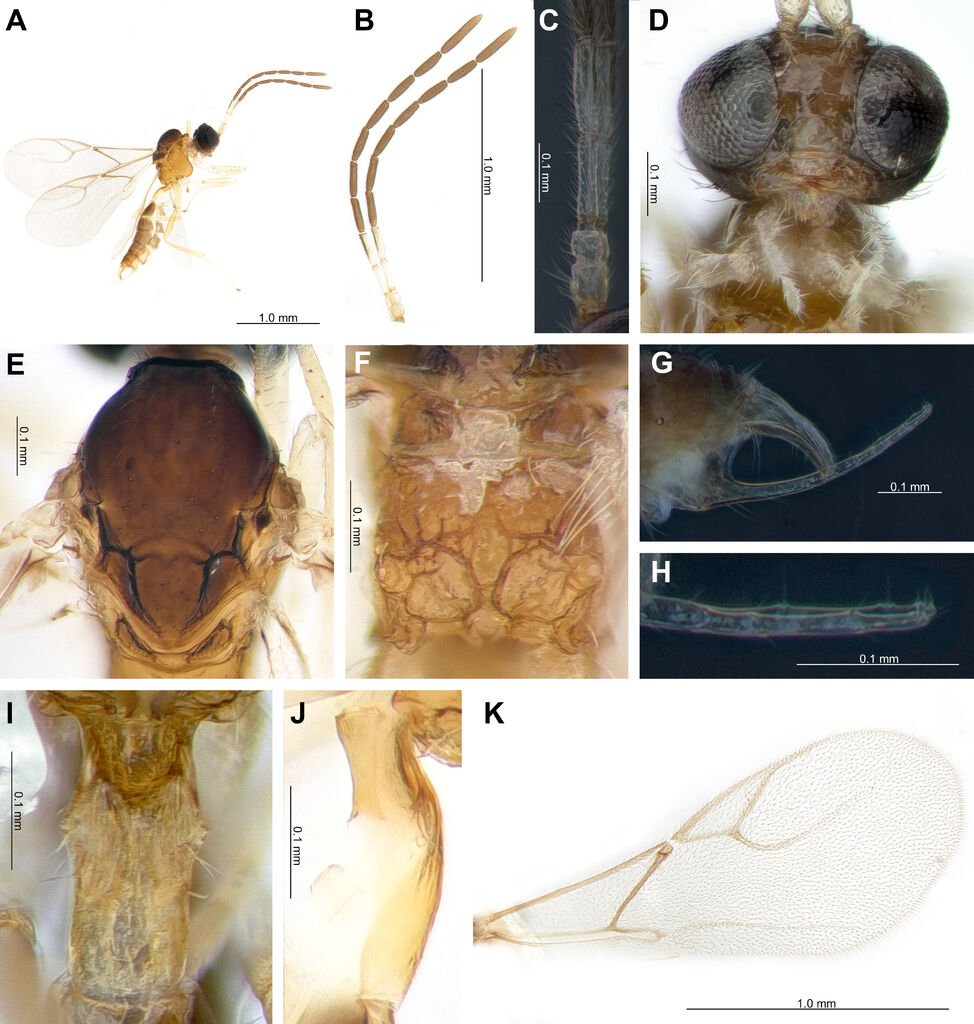
*Trioxysremaudierei*: **A** Habitus; **B** Antenna; **C** F1 and F2; **D** Head; **E** Mesoscutum; **F** Propodeum; **G** Ovipositor; **H** Prong; **I** Dorsal view of petiole; **J** Lateral view of petiole; **K** Forewing.

**Metasoma.** Petiole 1.5 times as long as wide at spiracles (Fig. 1I and J). Ovipositor sheath stout, concave on ventral margin. Ratio of ovipositor sheath width/length 2.0 at base (Fig. 1G). Anal prongs (= accessory prongs) almost straight, slightly curved upwards at apex. Dorsal side of prongs four setae, with one claw-like apical bristle and two setae at apex (Figure 2H).

**Colour.** Antenna brown; scape, pedicel and F1 yellowish-brown. Head and face dark brown, clypeus with mouth-parts yellowish-brown. Mesosoma and metasoma brown; Petiole yellowish-brown. Legs yellowish-brown with dark apices.

**Parasite of**: *Takecallistaiwana* on *Sasaborealis*

### Trioxys remaudierei

Starý & Rakhshani, 2017

BF6C6285-558C-5605-ABB4-58A92A4784D8

PP373118

PP373119

*Trioxysremaudierei* Starý & Rakhshani, 2017 - Rakhshani et al. (2017): 1237-1248.

#### Materials

- **Type status:** Other material. **Occurrence:** individualCount: 7; sex: female; lifeStage: adult; occurrenceStatus: present; preparations: in 80% ethanol, dry-specimen; occurrenceID: 98714FFC-467A-5148-97A4-D142E794F19F; **Taxon:** scientificName: Trioxy remaudierei Starý & Rakhshani, 2017; kingdom: Animalia; phylum: Arthropoda; class: Insecta; order: Hymenoptera; family: Braconidae; genus: Trioxys; specificEpithet: remaudierei; scientificNameAuthorship: Starý & Rakhshani, 2017; **Location:** higherGeography: East Asia; country: South Korea; countryCode: KR; stateProvince: Jeollabuk-do; municipality: Gimje-si; locality: Cheongha-myeon; verbatimLocality: 119, Jangsan-ri; **Identification:** identifiedBy: Sangjin Kim, JuHyeong Sohn, Hyojoong Kim; **Event:** eventDate: 07-11-2022; year: 2022; month: 11; day: 7; **Record Level:** language: en; institutionCode: KSNU

#### Description

**Female.** Length of body about 2.3 mm (Fig. 2A). Length of forewing 1.9 mm (Fig. 2K).

**Head.** Eyes oval, sparsely setose. Tentorial index 0.14 (Fig. 2D). Clypeus oval with seven setae. Malar space 0.08 times as long as longitudinal eye diameter. Antenna 11-segmented (Fig. 2B). F1 slightly shorter than F2 (F2 1.07 times as long as F1) (Fig. 2C). F1 and F2 5.0 and 4.1 times as long as their width at the middle, respectively. F1 without longitudinal placodes and F2 with 3-4 longitudinal placodes. Maxillary palp with four palpomeres, labial palp with two palpomeres. Ratio of eye to temple in dorsal view 0.6. Face width/height ratio 1.2 (Fig. 2D).

**Mesosoma.** Mesoscutum with notaulices on anterior part, dorsal surface smooth, two rows of scarcely setose start at end of notaulus (Fig. 2E). Head width/mesoscutum width ratio 1.3. Propodeum areolated, areola length/width ratio 1.2 (Fig. 2F). Pterostigma 3.5 times as long as width. Ratio of pterostigma length to R1 vein (= metacarpus) length 2.5 (Fig. 2K). r and RS veins extended.

**Metasoma.** Petiole 2.0 times as long as wide at spiracles (Fig. 2I and J). Ovipositor sheath elongate, gently concave on ventral margin. Ratio of ovipositor sheath width/length 2.8 at base (Fig. 2G). Anal prongs (= accessory prongs) long and straight upwards, bifurcated on apical one-third to two-thirds (apical one-third are four, apcial half is two, apical two-third is one), each having one claw-like apical bristle and one seta at apex with two or three setae at dorsal side (Fig. 2H).

**Colour.** Antenna brown; scape, pedicel, F1 and F2 yellowish-brown, sometimes dorsal part of F2 brown; Head, face and clypeus with mouth-parts dark brown. Dorsal side of mesoscutum and metasoma dark brown, except light brown propodeum and yellowish-brown petiole. Legs yellowish-brown with dark apices.

**Parasite of**: *T.arundinariae* on *Phyllostachysbambusoides*.

#### Notes

In the original description, the antenna of this species was 13-segmented. However, it was a mistake in the email from the author. This species is really 11-segmented, but two segments are artificially repeated in the line drawing.

## Analysis

**Figure 3.**
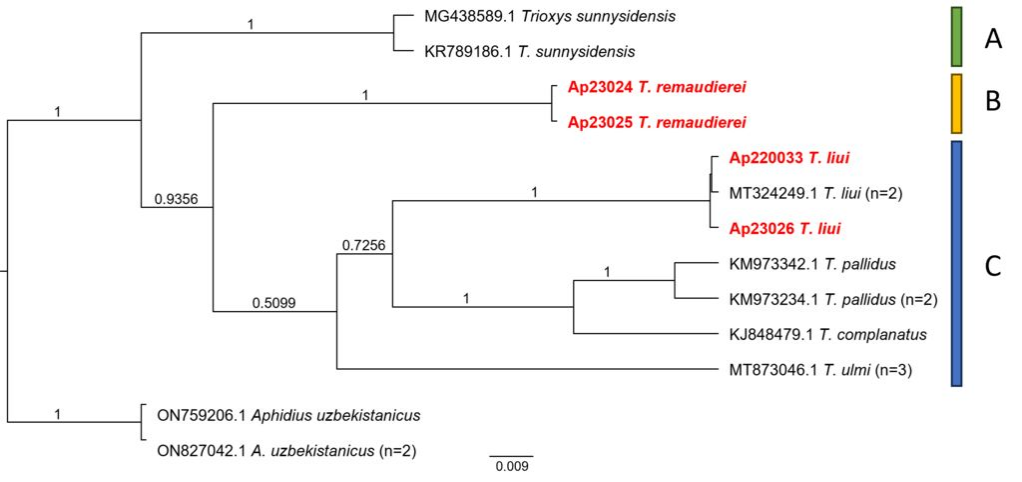
Phylogenetic tree of six *Trioxys* spp. estimated by the Bayesian method using their *COI* DNA barcode data. *Aphidiusuzbekistanicus* was used as an outgroup. Bootstrap support values more than 50% are indicated above branches. Scale-bar means the expected rate of a nucleotide substitution.

A Bayesian tree was constructed with twelve sequences from seven species, including an outgroup. Three clades were identified: Clade A represented *Trioxyssunnysidensis*, Clade B included *T.remaudierei*, both identified as monophyletic and the remaining species formed Clade C (Fig. 3). Within Clade C, *T.liui* was observed as the sister group to *T.pallidus*, *T.companatus* and *T.ulmi*. In comparison to a previous study by Čkrkić et al. (2021), *T.liui* exhibited a consistent pattern, but in this study, it was positioned between *T.ulmi* and the *T.pallidus-complanatus* group. *Trioxysremaudierei* was situated between Clade A and Clade C (Fig. 3).

Intraspecific and interspecific distances ranged from 0.000 to 0.011 (averaging 0.003) and 0.057 to 0.128 (averaging 0.108), respectively (Table 2). In Clade C, interspecific genetic distances ranged from 0.102 to 0.120 (averagint 0.113) between *T.liui* and the other species (*T.pallidus*, *T.complanatus* and *T.ulmi*). Genetic distances between *T.remaudierei* and *T.sunnysidensis* were 0.105 and genetic distances within Clade C were 0.111 (Table 2).

## Discussion

Most of the *Trioxys* species typically exhibit morphology characterised by two prongs (Mackauer 1960, Mackauer 1961). However, *T.remaudierei* stands out as it starts with a single prong that graudally bifucates into two towards the apex (Fig. 2G and H). This morphology is not unique to *T.remaudierei* alone, other species like *T.tenuicaudus* Starý, 1978, *T.udalovi* Davidian, 2005, *T.betulae* Marshall, 1896 and *T.artistigma* Telenga, 1953 also exhibit this mophology (Davidian 2005, Davidian 2007). The genus *Trioxys* uses anal prongs (= accessory prongs) for grasping and restraining host movement (Eidmann 1924, Schlinger and Hall 1961, Shaw and Huddleston 1991) and may have evolved into two strands to maximise it. Therefore, such prong morphology was considered a potential indicator of evolutionary ancestral traits even although it seemed to be not a clear correlation between taxa due to the limited number of samples in this study.

In case of *T.liui*, it is parasitic on *Cranaphisformosanus* (Takahashi) (Liu, 1975) and parasitic on *Phyllaphoidesbambusicola* Takahashi, 1921 on *Phyllostachysmakinoi* Hayata, 1915 in China (Chou and Chou 1993). Moreover, *Takecallis* sp. has been recorded on *Phyllostachysaurea* Carrière ex. Rivière & C. Rivière, 1878, *T.taiwana* and *T.arundinariae* on *Phyllostachys* sp. and captured on *Indocalamustessellatus* (Munro) Keng f., 1957 in Spain (Rakhshani et al. 2020). Similarly, *T.remaudierei* parasite *T.taiwana* on *Phyllostachys* sp. in France and *T.arundinariae* on *Phyllostachys* sp. in Spain (Rakhshani et al. 2017). This study newly records of *T.liui* is parasitic on *T.taiwana* on *Sasaborealis*, and *T.remaudierei* is parasitic on *T.arundinariae* on *P.bambusoides* in South Korea.

Both species have already been recorded in Europe and are considered invasive or subsequent adaptation into western Europe (Rakhshani et al. 2020). Aphidiinae species exhibit strong host specificity (Starý 1978) and both species show a strong host specificity for *Tacekallis* species. Futhermore, since *Takecallis* species have been recorded only on bamboo species (Lee and Lee 2018), the distribution of *T.liui* and *T.remaudierei* parasitising on it seems to be limited to areas where bamboo is located. In the natural range of *Tacekallis* species, all known species of this genus are restricted to East Asia (China, India, Japan, Korea, Manchurian subregion, Taiwan), *T.arundicolens* (Clarke, 1903), *T.arundinariae* and *T.taiwana*, *T.nigroantennatus* Wieczorek, 2023 are now widely distributed and introduced to other countries, including Africa (Algeria), USA (California), England, Netherlands, Hungary, Madeira, Australia, New Zealand, Argentina, Spain and Poland (Blackman and Eastop 2023, Wieczorek and Sawka-Gądek 2023). Consequently, parasitoids of *Takecallis* species are likely to be invasive species outside East Asia, with invasive countries potentially hosting distributions of these species.

## Supplementary Material

XML Treatment for Trioxys liui

XML Treatment for Trioxys remaudierei

## Figures and Tables

**Table 1.** Analysis sample list from GenBank (1-11, 16-18) with our own sample (12-15).

**No**	**Species**	**NCBI accession number**	**BOLD ID**
**1**	*Trioxyscomplanatus*	KJ848479.1	-
**2**	*T.liui*	MT324249.1	-
**3**	*T.liui*	MT324250.1	-
**4**	*T.pallidus*	KM973397.1	GBAHB12494-15
**5**	*T.pallidus*	KM973342.1	GBAHB1349-15
**6**	*T.pallidus*	KM973234.1	GBAHB1457-15
**7**	*T.sunnysidensis*	KR789189.1	JSJUN008-11
**8**	*T.sunnysidensis*	MG438589.1	BARSE352-16
**9**	*T.ulmi*	MT873046.1	-
**10**	*T.ulmi*	MT873047.1	-
**11**	*T.ulmi*	MT873048.1	-
**12**	*T.liui*	PP373116.1	-
**13**	*T.liui*	PP373117.1	-
**14**	*T.remaudierei*	PP373118.1	-
**15**	*T.remaudierei*	PP373119.1	-
**16**	*Aphidiusuzbekistanicus*	ON759206.1	-
**17**	*Aphidiusuzbekistanicus*	ON827042.1	-
**18**	*Aphidiusuzbekistanicus*	ON827045.1	-

**Table 2.** Calculated genetic distances, based on COI sequences between *Trioxys* spp. used in the analysis.

	*T.liui* (n = 4)	*T.remaudierei* (n = 2)	*T.complanatus*	*T.pallidus* (n = 3)	*T.sunnysidensis* (n = 2)	*T.ulmi* (n = 3)
*Trioxysliui*	(0.000)	-	-	-	-	-
*T.remaudierei*	0.111	(0.000)	-	-	-	-
*T.complanatus*	0.120	0.120	(0.000)	-	-	-
*T.pallidus*	0.118	0.113	0.057	(0.011)	-	-
*T.sunnysidensis*	0.110	0.105	0.121	0.128	(0.005)	-
*T.ulmi*	0.102	0.101	0.115	0.106	0.100	(0.000)
